# Life Satisfaction and Dental Visits in Adults Aged ≥50 Years and Living with Diabetes Mellitus: A Comparison between Urban and Rural Mexican

**DOI:** 10.1155/2023/5499990

**Published:** 2023-07-31

**Authors:** Alvaro García Pérez, Juan Carlos Cuevas-González, Teresa Villanueva Gutierrez

**Affiliations:** ^1^Faculty of Higher Studies (FES), Iztacala, National Autonomous University of Mexico (UNAM), Mexico; ^2^Stomatology Department, Biomedical Sciences Institute, Autonomous University of Ciudad Juárez, Ciudad Juárez 32310, Mexico; ^3^Health Care Department, Metropolitan Autonomous University-Xochimilco, Mexico

## Abstract

**Objective:**

To examine the association between low life satisfaction with past-year dental visits in a rural-urban national sample of 50-year-old Mexican adults with diabetes mellitus (DM).

**Methods:**

Data are drawn from the Mexican Health and Aging Study (MHAS), a cross-sectional study conducted in 2018 involving 3,592 older adults aged 50 years and older and living in urban and rural areas in Mexico. Life satisfaction was measured using the Satisfaction with Life Scale (SWLS) and past-year dental visits dichotomized as none and ≥1 dental visits. The Poisson regression analyses were used to assess the association, adjusting for confounders.

**Results:**

62.9% were women, mean age was 65.5 (±9.6), and 16.5% lived in a rural area, while the female subjects continue to present a higher probability of visiting a dentist (PR = 1.28 (95% CI 1.08–1.51)). In terms of age, the ≥70-year group presented 28% lower possibility of visiting a dentist (PR = 0.72 (95% CI 0.60–0.86)). The interaction showed that adults ≥50 years who reside in a rural area and have low life satisfaction were 40% less likely to have visited a dentist in the last year (PR = 0.60 (95% CI 0.37–0.98)) than adults ≥50 years who reside in an urban area and have high life satisfaction.

**Conclusions:**

The present study highlights the association between low life satisfaction and past-year dental visits in rural populations. Therefore, rurality should be considered a possible confounder in analysis of life satisfaction in the older adult population.

## 1. Introduction

According to statistics obtained from the International Diabetes Federation, by 2021, the approximate prevalence of DM was 10.5% for a population of 537 million [1], rising to 11.3% by 2030 for a population of 643 million people and, by 2045, to 12.2% for a population of 783 million people [1]. Globally, in 2021, the prevalence of DM in urban and rural areas was 12.1% and 8.3%, respectively, while, by 2045, the prevalence in urban areas is projected to rise to 13.9% as a result of an aging population [1]. In Mexico, according to the Encuesta Nacional de Salud y Nutrición 2018 and 2020 (ENSANUT, or the National Health and Nutrition Examination Survey), the prevalence of diabetes was 16.8% in 2018 and 15.7% in 2020 [2].

The macro- and microvascular complications caused by DM have adverse effects not only on general health but also on long-term oral health, leading to increases in the number of medical appointments and the consumption of medication. The multiple complications resulting from DM require prolonged hospitalizations, exerting both a physical and emotional impact on patients and negatively impacting personal and familial wellbeing [3]. Furthermore, DM can affect the patient's state of mind and self-esteem, thus negatively affecting their quality of life and life satisfaction [4].

Life satisfaction involves the subjective evaluation of the individual and has been identified as a part of subjective wellbeing, which can change at any time. It is an important component of psychological adaptation and a healthy aging process and can even be an indicator of adaptation to various changes and the reality of aging [5]. Studies have shown a relationship between predictive variables and life satisfaction, with Borg et al., finding that perception of health has a significant effect on life satisfaction [6]. In contrast, Fernández-Ballesteros et al. reported negative effects of physical diseases on life satisfaction [7]. It has been shown that DM is associated with a reduction in both happiness and life satisfaction [8], while previous studies have found an association between changes to perceived health and life satisfaction in DM patients [9]. Both emotional anguish and cognitive function showed significant negative correlations with life satisfaction in DM patients [4].

Various studies have reported that people with DM experience a greater frequency of oral complications, such as caries, xerostomia, tooth loss, and periodontal disease [10, 11]. Similarly, in older adults in Mexico, there have been reports prevalence of various oral health indicators such as edentulism 38.9%, dental caries in 95.3%, and the prevalence of severe periodontitis that was 80%. These conditions accumulate throughout the life and are exacerbated by infrequent dental visits, compared with the general population [12, 13]. It has been observed that older adults who frequently visit the dentist present a positive impact in terms of the prevention of oral diseases. Studies have shown that 46% of older adults in the United States [14] and 14.0% of older adults residing in rural areas in Mexico have visited the dentist [15], while 40.6% of DM patients in Mexico with health insurance have visited the dentist [16].

While few studies have researched life satisfaction in relation to dental visits in adults, Valdez et al. found that frequent dental visits are associated with lower life satisfaction in adults ≥40 years [17]. However, these conclusions are based on research that included adults ≥40 years and did not analyze related factors, such as pertaining to a rural population or having DM. Life satisfaction indicates the subjective wellbeing which is related with the health and mortality status among the older adults. On the other hand, the perception that a person has about their state of health will have a fundamental role during aging process, since it involves subjective experience that they have about their current state of health. In addition, the relationship between perception of health and life satisfaction in older adults has been explored, since it has been reported that this last variable has a significance in maintenance and prediction of general health [18].

Given the foregoing, greater knowledge is required on the factors contributing to life satisfaction and dental visits in older adults. This information can be used to develop diagnostic, prevention, and intervention strategies for helping older adults with DM to maintain and increase both the level of life satisfaction and the frequency of dental visits. Therefore, the present study is aimed at examining the association between low life satisfaction and past-year dental visits in a rural-urban national sample of 50-year-old Mexican adults with DM. We hypothesized that adults ≥50 years with diabetes who live in a rural area will have a lower chance of visiting a dentist in the last year compared to population living in urban areas.

## 2. Methods

The Mexican Health and Aging Study (MHAS) is a nationally representative prospective panel study designed to examine the influence of diseases on the health, functionality of life, and life span of the Mexican population aged 50 and over from both urban and rural areas. The surveys are conducted under the supervision of coordinators from both Mexico and the United States, while MHAS is partially funded by the National Institutes of Health/National Institute of Aging and the Instituto Nacional de Estadística y Geografía (INEGI or National Institute of Statistics and Geography). The data files and documentation are available for public use at https://enasem.org/Home/index_esp.aspx. The thematic content of the MHAS survey included the following: demographic data, the number of residents of a household and children rosters, self-reported health in various dimensions (chronic diseases, physical function, perceived global health, depression, and cognition), institutional support, life satisfaction, use of time, social support and social engagement, housing conditions, and economic aspects, such as health expenditure, health insurance coverage, pensions received or expected, income by sources, and the value of accumulated assets.

### 2.1. Ethics Approval and Consent to Participate

The research carried out by the present study on the MHAS databases adhered to the relevant ethical criteria for research on human subjects and were approved by the corresponding ethics committee (University of Texas Medical Branch and, in Mexico, INEGI and the National Institute of Public Health) (NIH R01AG018016). Informed consent was obtained in writing from all participants.

### 2.2. Study Design

The present study adhered to a cross-sectional model.

### 2.3. Study Population

Of the sample of 4,193 individuals who reported having diabetes in MHAS-2018, 601 were excluded due to missing responses, with the final sample comprising 3,592 diabetic adults aged 50 years and older. No differences were observed in the demographic data pertaining to the adults aged 50 and over in terms of missing responses and responses received. The inclusion criteria applied by the present study for participants were patients over 50 years of age, who were of either sex, and who did not present missing data in the database. The exclusion criteria were participants who did not sign informed consent or who refused to participate in the study.

Adult subjects were classified as having prevalent self-reported diagnosed diabetes (hereafter referred to as diabetes mellitus) if they answered “yes” to the question, “Has a doctor or another medical practitioner ever diagnosed you with diabetes?”

### 2.4. Independent Variable: Life Satisfaction

The present study used the SWLS, a questionnaire included in the Spanish version of the MHAS 2018 and which measured subjective criteria for life satisfaction that correspond to five items evaluated via multiple-choice options. The MHAS academic committee discussed the need to adapt the response scale of the original SWLS for use in the Mexican population and decided to reduce the response options to three choices: 1 = agree, 2 = neither agree nor disagree, and 3 = disagree. The scores ranged from 1 to 3, while the possible scores for the adapted SWLS scale ranged from 5 (high satisfaction) to 15 (low satisfaction) [19]. High values on the SWLS reflect low life satisfaction.

### 2.5. Dependent Variable: Past-Year Dental Visits

The older adults were classified according to their responses to the following question: “In the last year, how often… have you seen a dentist?” They were then categorized into two groups—none and ≥1 dental visits.

### 2.6. Covariates

The following sociodemographic variables were used as potential confounders and adjusted for the models applied: age (in years), categorized into three groups (50-59 years, 60-69 years, and ≥70 years); sex (men/women); place of residence (urban/rural); smoking status, categorized into three groups (never/current/former smoker); alcohol consumption, categorized into three groups (yes/no/has never drunk alcohol); and years of education, which was used to compare those adults who had completed nine years of formal education or more, with those who had completed less than nine years (corresponding in Mexico to primary and secondary school combined), categorized into three groups (no education, 1-9 years, and ≥10 years). To assess the impact of stress on health, the following question was used: “In the last 12 months, how much do you think that stress has affected your health?” The results were categorized into two groups (very much/something and almost nothing/nothing).

### 2.7. Statistical Analysis

All analyses were conducted using Stata 15 software (Stata Corp, College Station, TX, USA). Cross-tabulations using Pearson's Chi-square test were used to describe associations between past-year dental visits and age group, sex, years of education, place of residence (rural/urban), smoking status, alcohol consumption, stress-related health issues, low life satisfaction, and place of residence (rural/urban). Multivariable analysis (Poisson's regression with robust variance) was used to determine whether past-year dental visits were associated with low life satisfaction, after controlling for other characteristics. The choice of this method of regression is justified because of the high frequency of visits to a dentist in the past year. The dependent variable was having undertaken a dental visit in the past year (discrete variable). All independent variables were categorical (age, sex, residence, years of education, smoking, and stress-related health issues). In the analysis conducted, the life satisfaction scale was dichotomized into an SWLS score 3rd quartile (SWLS < 9 = 0 (high) and SWLS ≥ 9 = 1 (low)), with the results compared in terms of the prevalence ratios (PRs) and respective 95% CIs. Interaction terms were introduced for years of education and low life satisfaction and place of residence and low life satisfaction, while their significance was tested via a heterogeneity test. *p* values < 0.05 in a two-tailed test were considered statistically significant.

## 3. Results

### 3.1. Population Characteristics

Of the total number of participants with diabetes, 63.0% were women (2261), and 37.0% (1331) were men, giving a mean age of 65.5 (±9.5) years. Furthermore, 84.5% (3034) of participants had ≤9 years of education, and 16.5% (594) lived in a rural area. Of the total sample, the percentage of past-year dental visits was 40.8% (1464). Dental visits in the past year were associated with place of residence. The urban population reported a higher percentage of dental visits than the rural population (42.7% vs 30.8%; *p* < 0.001, respectively), while 41.4% of the rural population presented low life satisfaction compared to 35.0% of the urban population (*p* = 0.003) (Table 1).

**Figure 1:**
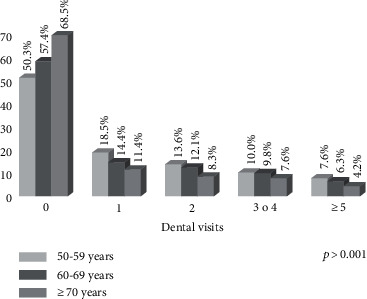
Percent distribution of past-year dental visits by age group in adults ≥50 years with diabetes in Mexico.

In each group for age, gender, education, tobacco and alcohol use, and stress-related health issues, the proportion of dental visits in the last year were similar for both the urban and rural population (Table 2). In the gender categories, tobacco and alcohol use and stress-related health issues did not influence the dental visits, while, in contrast, advanced age was strongly related to a lower percentage of participants who had visited the dentist in the last year, for both the urban and rural populations.  Figure 1 shows the same trend in terms of the frequency of dental visits in the last year per age group of adults with diabetes.

### 3.2. Multivariable Analysis

The results of the application of the Poisson regression analysis can be seen in Table 3. After adjusting for other variables, women continue to present a higher possibility of visiting a dentist (PR = 1.28 (95% CI 1.08–1.51)), while stress-related health issues were positively associated with dental visits. In terms of age, the ≥70-year group presented a 28% lower possibility of visiting a dentist (PR = 0.72 (95% CI 0.60–0.86)), while the lower the number of years of education, the lower the possibility of visiting a dentist. Moreover, the interaction between rural location and low life satisfaction (PR = 0.60 (95% CI 0.37–0.98)) was significant. The interaction observed showed that the subjects from a rural area and reporting low life satisfaction presented a 40% lower possibility of having visited a dentist in the last year than subjects from an urban area and reporting high life satisfaction.

## 4. Discussion

Based on a nationally representative sample, the results obtained by the present study show the inequalities in the use of dental care between urban and rural areas in Mexico. Adults with DM ≥50 years old with low life satisfaction and living in rural areas presented a 40% lower possibility of visiting the dentist than those residing in urban areas. DM is a chronic metabolic disease that can directly affect not only the subject's state of health but also their independence and functionality of life, further to reducing their subjective wellbeing. Studies have shown that people with DM present lower life satisfaction than the general population [20, 21]. Moreover, as a longer duration of DM has been associated with reduced subjective wellbeing [22], it is necessary to search for psychological factors that may help adults with DM to improve their life satisfaction.

In Mexico, a gradual aging process has been observed in the population, where, in 2020, according to INEGI, nearly 28 million people were aged 50 years or over, of whom approximately six million lived in rural communities. Notable among the factors significantly influencing the differences between rural and urban environments are economic, social, and psychological factors, as well as the life conditions related to social class, access to healthcare services, and the presence of chronic diseases, all of which can impact life satisfaction [23, 24]. For example, in a population of older adults in Hong Kong, poverty was found to reduce life satisfaction [25]. In contrast, having a high educational level and being in paid employment increased life satisfaction in older adults [26]. Li et al. found that the life satisfaction reported by older Chinese adults living in rural areas was associated with educational level, financial resources, and self-evaluated health [27]. Therefore, not only should the determinants associated with the presence of DM in older adults but also the ideas, expectations, and feelings produced by the disease be taken into account, as should the particular context of each person, in order to generate a positive impact on life satisfaction in both urban and rural areas.

While access to healthcare services is fundamental to good health, the rural population encounters barriers that reduce its capacity to access the medical attention it needs. The geographical location of these rural locations limits the permanent presence of healthcare professionals and, therefore, restricts access to continuing healthcare [28]. For example, Salinas et al. reported that lack of health insurance coverage was associated with a lower probability of visiting a doctor in older Mexican adults living in rural areas [29]. Similarly, older adults living in rural areas in Mexico and Australia report a lower probability of visiting a dentist than the corresponding urban population (34% and 14%, respectively) [15, 30]. In contrast, it has been observed that, in the last 30 years, the rates of healthcare coverage increased from 30% to nearly 50% in adults with DM and living in rural areas in Mexico [31]. Therefore, the rural population is a more vulnerable group, due to the fact that access to basic services such as education, healthcare, and housing represent, for them, conditions of social disadvantage that impede them in accessing limited healthcare services.

Few studies have evaluated the association between the level of life satisfaction and dental visits in adults with DM. For example, the results of the recent fifth wave of the German Ageing Survey conducted on adults ≥40 years found that frequent dental visits are related to low life satisfaction. Despite being a nationally representative sample, the survey did not specify whether it included patients with DM [17]. On the other hand, Ekbäck et al., in a cohort of adults ≥50 years, did not find significant changes in/between the level of satisfaction and dental care [32]. The present study found that adults with DM, ≥50 years old, with low life satisfaction, and living in rural areas presented a 40% lower probability of visiting the dentist than the corresponding population living in urban areas.

Although, normally, it is thought that life satisfaction is relatively constant in the long term, it can change in response to life circumstances, in that some people adapt more easily to new situations, and others present negative or positive changes [33]. A significant finding of the present study was the relationship observed between low life satisfaction and fewer dental visits, which may be due to the manner in which the older adult perceive themselves, which is one of the multiple psychological aspects that may change life satisfaction when a person moves into their third age [34]. Depending on other factors, such as age, gender, civil status, education, and income, older adults' life satisfaction varies; therefore, when the basic needs of an individual are not met, it is difficult for them to be satisfied with life.

The present study found that women with DM are 28% more likely to visit the dentist than men.

Research shows that women present better oral hygiene, a lower occurrence of tooth loss due to caries, a lower occurrence of periodontal disease, and a better self-evaluation of oral health [35]. These findings are due to the fact that women have clearer and more healthy beliefs about their oral and general health and, furthermore, are more likely than men to believe that oral health and oral health prevention have a greater positive impact on both their appearance and wellbeing [34].

Finally, one of the findings of the present study was that, as age increased and level of education decreased, the probability of adults with DM and ≥50 years visiting the dentist in the last year also decreased. In fact, longitudinal studies have found that older adults use dental services less frequently than young adults [36].

Supervising the treatment of DM in rural populations is a complex task due to factors that exacerbate inequalities in access to healthcare services and cause, as a consequence, decreased life satisfaction. The *Instituto Nacional de Salud para el Bienestar* (INSABI or National Health Institute for Welfare) is responsible for reducing this health equality gap between the rural and urban population in Mexico. One of its objectives is to ensure the provision of universal free medical attention and tackle the gaps both in healthcare coverage between urban and rural areas and in the availability of medical staff in Mexico's most remote and marginalized regions.

## 5. Limitations

One of the limitations of the present study is the fact that its findings are based on cross-sectional data, which do not allow for causal inference, while another is the use of the self-reported questionnaire to evaluate DM as a substitute for a confirmed clinical diagnosis. However, self-reported DM has been reported with a good level of reliability. On the other hand, one of the advantages of the present study is that the sample was representative, for both urban and rural areas, of adults aged 50 years or over in Mexico, thus enabling the evaluation of the impact of chronic disease and aging-related factors on the use of dental services by the population.

## 6. Conclusions

Adults with DM aged ≥50 years with low life satisfaction and residing in rural areas presented a 40% lower probability of visiting the dentist than those residing in urban areas. Therefore, research conducted in this area should consider rurality as a possible confounder in analysis evaluating life satisfaction; moreover, it is necessary to identify the factors that may increase life satisfaction in third age.

## Figures and Tables

**Table 1:** Sample characteristics in adults ≥50 years in urban and rural with diabetes in Mexico (*n* = 3,592).

	Population		*p* ^∗^
	Urban (*n* = 2998) *n* (%)	Rural (*n* = 594) *n* (%)	
Age			
50–59 years	567 (26.6)	560 (38.2)	<0.001
60–69 years	659 (31.0)	489 (33.4)	
≥70 years	902 (42.4)	415 (28.4)	
Sex			
Men	799 (37.6)	532 (36.3)	0.461
Women	1329 (62.4)	932 (63.7)	
Years of education			
No education	374 (17.6)	126 (8.6)	<0.001
1-9 years	1533 (72.0)	1001 (68.4)	
≥10 years	221 (10.4)	337 (23.0)	
Smoking status			
Never	1476 (69.4)	1025 (70.0)	0.070
Current	171 (8.0)	143 (9.8)	
Former smoker	481 (22.6)	296 (20.2)	
Alcohol consumption			
No	1411 (66.3)	926 (63.2)	0.001
Yes	427 (20.1)	392 (26.8)	
Never drank alcohol	290 (13.6)	146 (10.0)	
In the last 12 months, how much effect do you think stress has had on your health?			
Very much/something	1391 (65.4)	844 (57.6)	<0.001
Almost nothing/nothing	737 (34.6)	620 (42.4)	
Life satisfaction (SWLS)			
SWLS <9	1949 (65.0)	348 (58.6)	0.003
SWLS ≥9	1049 (35.0)	246 (41.4)	
Past-year dental visits			
None	1717 (57.3)	411 (69.2)	<0.001
≥1	1281 (42.7)	183 (30.8)	

^∗^Chi-square test.

**Table 2:** The percentage of adults ≥50 years who visited a dentist in the past year by urban and rural zone with diabetes in Mexico (*n* = 3,592).

	Past-year dental visits					
	Urban population			Rural population		
	None (*n* = 1717) *n* (%)	≥1 (*n* = 1281) *n* (%)	*p* ^∗^	None (*n* = 411) *n* (%)	≥1 (*n* = 183) *n* (%)	*p* ^∗^
Age						
50–59 years	444 (25.9)	483 (37.7)	<0.001	123 (29.9)	77 (42.1)	0.004
60–69 years	522 (30.4)	428 (33.4)		137 (33.3)	61 (33.3)	
≥70 years	751 (43.7)	370 (28.9)		151 (36.8)	45 (24.6)	
Sex						
Men	662 (38.6)	476 (37.2)	0.436	137 (33.3)	56 (30.6)	0.512
Women	1055 (61.4)	805 (62.8)		274 (66.7)	127 (69.4)	
Years of education						
No education	261 (15.2)	85 (6.6)	<0.001	113 (27.5)	41 (22.4)	0.001
1-9 years	1247 (72.6)	877 (68.5)		286 (69.6)	124 (67.8)	
≥10 years	209 (12.2)	319 (24.9)		12 (2.9)	18 (9.8)	
Smoking status						
Never	1150 (67.0)	880 (68.7)	0.048	326 (79.3)	145 (79.2)	0.982
Current	150 (8.7)	133 (10.4)		21 (5.1)	10 (5.5)	
Former smoker	417 (24.3)	268 (20.9)		64 (15.6)	28 (15.3)	
Alcohol consumption						
No	1146 (66.7)	814 (63.6)	<0.001	265 (64.5)	112 (61.2)	0.131
Yes	360 (21.0)	350 (27.3)		67 (16.3)	42 (23.0)	
Never drank alcohol	211 (12.3)	117 (9.1)		79 (19.2)	29 (15.8)	
In the last 12 months, how much effect do you think stress has had on your health?						
Very much/something	1120 (65.2)	729 (56.9)	<0.001	271 (65.9)	115 (62.8)	0.465
Almost nothing/nothing	597 (34.8)	552 (43.1)		140 (34.1)	68 (37.2)	

^∗^Chi-square test.

**Table 3:** Adjusted prevalence ratios (PR) from Poisson's regression analysis of the number of visits to dentist among adults aged ≥50 years with diabetes in Mexico (*n* = 3,592).

	Crude PR (95% CI)	*p*	Adjust PR (95% CI)	*p*
Sex				
Men	Reference		Reference	
Women	1.17 (1.10–1.25)	<0.001	1.28 (1.08–1.51)	0.004
Age				
50–59 years	Reference		Reference	
60–69 years	0.80 (0.74–0.85)	<0.001	0.91 (0.75–1.09)	0.321
≥70 years	0.55 (0.51–0.59)	<0.001	0.72 (0.60–0.86)	<0.001
Years of education				
≥10 years	Reference		Reference	
1–9 years	0.52 (0.48–0.55)	<0.001	0.56 (0.45–0.71)	<0.001
No education	0.28 (0.25–0.32)	<0.001	0.37 (0.27–0.51)	<0.001
Life satisfaction				
SWLS < 9 (high)	Reference		Reference	—
SWLS ≥ 9 (low)	0.37 (0.34–0.40)	<0.001	0.42 (0.34–0.51)	
Place of residence				
Urban	Reference		Reference	
Rural	0.59 (0.54–0.65)	<0.001	0.78 (0.63–0.96)	—
Rural locality and low life satisfaction (SWLS ≥ 9) (interaction terms)	—	—	0.60 (0.37–0.98)	0.042
Smoking				
No	Reference		Reference	
Yes	1.18 (1.07–1.31)	0.001	1.07 (0.79–1.45)	0.650
Stress-related health				
Almost nothing/nothing	Reference		Reference	
Very much/something	1.36 (1.28–1.44)	<0.001	1.21 (1.03–1.40)	0.014

RR: rate ratio, CI: confidence interval. Log likelihood = −6813.2679.

## Data Availability

The data that support the findings of this study are openly available in the Mexican Health and Aging Study (MHAS) at https://enasem.org/Home/Index.aspx, reference number NIH R01AG018016.
